# Interventions to Reduce Exposures in the Workplace: A Systematic Review of Intervention Studies Over Six Decades, 1960–2019

**DOI:** 10.3389/fpubh.2020.00067

**Published:** 2020-03-09

**Authors:** Johan Ohlander, Hans Kromhout, Martie van Tongeren

**Affiliations:** ^1^Institute for Risk Assessment Sciences, Utrecht University, Utrecht, Netherlands; ^2^Centre for Occupational and Environmental Health, School of Health Sciences, Faculty of Biology, Medicine and Health, University of Manchester, Manchester Academic Health Science Centre, Manchester, United Kingdom

**Keywords:** occupational exposure, biological exposure, chemical exposure, effectiveness, overview, occupational

## Abstract

**Background:** Reducing occupational ill-health from chemical and biological agents is realized primarily through the mitigation and elimination of hazardous exposures. Despite evidence of declining exposure in European and North-American workplaces, comprehensive studies of the effectiveness of workplace interventions for reducing hazardous exposure and associated work-related ill-health seem rare. We reviewed occupational intervention studies targeting exposure to chemical and biological agents, and determined trends in frequency and quality of such studies.

**Methods:** We searched Embase, Medline, and Web of Science for peer-reviewed original articles on occupational intervention studies published 1960–2019, aimed at reducing workers' exposure to dusts, gases, fumes, or liquids of chemical, biological, or mineral nature, or workers' risks for associated health outcomes. The frequency of articles, intervention types, intervention endpoints, and study quality of published intervention studies between 1960 and 2019 and according to 10-year intervals were analyzed.

**Results:** Of 3,663 retrieved articles, 146 intervention studies were identified and reviewed, of which 63 concerned control measures, 43 behavioral change, 28 use of personal protective equipment, and 12 workplace policies. Intervention endpoints were occupational exposures (73%), health outcomes (22%), and a combination of both (5%). Of reviewed studies, 38% involved a control group, 16% randomized the intervention, 86% were planned interventions, and 86% compared exposure or health outcomes pre and post intervention. Over time the number of intervention studies identified in this search increased from none during 1960–1969 to ~60 during 2000–2009 and 2010–2019, respectively. The study quality improved over time, with no studies during 1960–1989 that complied with the highest quality criteria. During 2000–2009 and 2010–2019 16 and 12% of studies, respectively, were judged to be of highest quality.

**Conclusion:** Despite an improvement over the last six decades in the frequency and quality of intervention studies targeting exposure to chemicals and biological agents, the absolute number of intervention studies remains low, particularly when considering only high quality studies. Occupational exposure to chemical and biological agents is still causing excessive disease in workforces worldwide. To reduce occupational ill-health caused by these exposures, it is important to expand the evidence on (cost-)effectiveness and transferability of interventions to reduce exposure and health effects.

## Introduction

Prevention and reduction of work-related ill-health from hazardous agents is realized primarily through the mitigation or elimination of exposures to such agents in the workplace. Knowledge about effective control of exposures to reduce adverse health effects is therefore key. There is undoubtedly a large amount of practical knowledge and experiences to reduce hazardous exposures amongst experts such as occupational hygienists and other health and safety experts.

Nevertheless, specific and comprehensive evidence on effectiveness and cost-effectiveness of interventions aiming to reduce exposure and improve health of workers from workplace intervention studies appears to be limited. Fransman et al. (1) reviewed in 2008 articles in the occupational hygiene field for the effectiveness of control measures such as local exhaust ventilation systems, and developed a database covering published evidence they located. The evidence was generally poor and often generated from cross-sectional exposure surveys that investigated the relationship between presence of control measures and levels of exposure. To generate high quality evidence of effectiveness, intervention studies should incorporate study design elements that minimize bias, by involving a control group, and measure pre and post intervention exposures or associated health outcomes (2). Additionally, workers, groups of workers, or workplaces should ideally be assigned randomly to the intervention and control groups, although natural experiments (i.e., studies where the researchers have no control over the intervention and the method of its implementation) can also provide very useful evidence.

Although a decreasing number of publications on occupational diseases the last 60 years is documented (3), a growing attention toward occupational intervention studies has been seen during the last decades (2). The implementation of the National Occupational Research Agenda (NORA) in the USA in the 1990s aimed amongst others to stimulate intervention effectiveness research (4). Generally, occupational interventions have focused on musculoskeletal disorders, stress, nutrition, and ergonomics (5). However, the progress and development regarding interventions targeted specifically toward exposure to chemical and biological agents and their effectiveness is less clear. A recent review of articles on Occupational Safety and Health (OSH) interventions published between 1966 and 2017 showed a strong focus on injuries, and musculoskeletal disorders (6) with only 5 of 45 reviewed interventions (7–11) specifically focused on chemical or biological exposures. Certainly, there is evidence that chemical and biological occupational exposures in some industries and sectors in Europe and North America have reduced over time, and incidence of associated occupational diseases subsequently have been reduced partly as a result of successful interventions. For example, coal workers' pneumoconiosis in the UK has declined through the closing of all mines during the 1980-ies, and through banning smoking in public places workers' exposure to second hand smoke has been significantly reduced. Some reviews of chemical and biological exposures suggest a similar declining time trend. Creely and colleagues found annual declines of up to 32% regarding inhalation exposure to aerosols, gases and vapors, and fibers (12), and Park et al. showed that exposures to metal working fluids declined from about 5.4 mg/m^3^ before 1970 to about 0.5 mg/m^3^ in 1990 (13). Vermeulen et al. (14) showed that control measures implemented over a nine-year period in seven rubber-manufacturing companies in the Netherlands led to an annual reduction of exposure concentrations by 5.7 and 6.7% for inhalable particulate and dermal exposure to cyclohexane soluble matter, respectively. However, there are also a number of exceptions. van Tongeren et al. evaluated levels of flour dust by occupation in the UK through extracting all flour dust data from the National Exposure DataBase (NEDB). The analysis showed that levels of flour dust exposure in the UK were high with an overall mean ranging from 7.8 mg/m^3^ in bakeries to 17.9 mg/m^3^ in flourmills. Moreover, the analysis did not support any downward trend in levels of flour exposure over the time period studied (15). In addition, some recent data suggest that this downward trend in exposure and incidence in occupational disease may be plateauing. For example, a recent analysis of data from The Health and Occupational Reporting network (THOR) suggested a possible reversal of the downward trend in the incidence of occupational asthma in the UK (16). For other hazardous agents there is evidence that health impact is still very large, despite reductions in exposure. For example, in relation to exposure to respirable crystalline silica, there is evidence that despite the introduction of new exposure limits, such as the new EU exposure limit of 100 μg/m^3^, exposures are still predicted to cause several hundred thousands of deaths in Europe by 2060 if no new interventions are being introduced (17). Consequently, it is clear that more work is needed to reduce exposures further.

To ensure that interventions are effective, there is a demand for well-conducted intervention studies that expand the present body of evidence. The aim of this review was to create an overview of occupational scientific literature describing interventions implemented at the workplace targeting occupational exposure to chemical and biological agents and the risk for associated health outcomes. We also determined trends in the quantity and quality of reviewed studies.

## Methods

We searched Embase (Embase.com), Medline (Ovid.com) and Web of Science (webofknowledge.com) for peer-reviewed original articles on occupational intervention studies targeting selected chemical and biological agents, published from 1960 to March 2019 in English, French, German, Spanish, Dutch, Swedish, Danish, and Norwegian. To allow for a review of different types of intervention studies with various levels of quality we included studies with different designs, ranging from studies evaluating intervention effects using cross-sectional designs, to randomized controlled trials.

### Search Terms

We used the following search terms applied as keywords and subject headings: occupational exposure, occupational safety, environmental exposure, chemical exposure, organic dust, dust, vapor, fiber, fume, and microorganism, combined with search terms reflecting eligible study types; controlled and uncontrolled before-after studies, randomized controlled trials (individual or cluster based), time-series analyses, and studies on effectiveness of personal protective equipment (PPE) or control measures (Supplementary File 1). Truncations of keywords were used to capture articles that use variations of applied keywords.

### Article Selection

Screening of articles was carried out in two rounds. Firstly, title and abstract screening of retrieved articles was performed by the lead author of this manuscript (J.O). This initial screening was carried out to exclude articles that were clearly ineligible. Secondly, remaining articles were assessed for eligibility by three reviewers (J.O, H.K, M.v.T). We included occupational intervention studies implemented at the workplace aimed at reducing workers' exposure to dust, gas, fumes, or liquids of chemical, biological, or mineral nature. We also included intervention studies which evaluated the risk for health outcomes associated with eligible exposures given that the exposure was described in the article. Further, studies which evaluated knowledge, awareness and practical skills associated with eligible exposures or with outcomes associated with eligible exposures were also included. We excluded (systematic) reviews, case reports, impact assessment studies (e.g., estimations of predicted future impact of interventions), conference abstracts, laboratory based studies/experimental studies (not executed in the workplace), clinical trials, clinical interventions, studies evaluating musculoskeletal health, or ergonomic risk factors, mental health or stress, and studies that specifically evaluated exposure to viruses or parasites. We also excluded studies which did not specifically describe the exposure being intervened on, e.g., studies which used composite measures or specific points associated with several exposures (18). The final selection of articles was stored in Endnote for further analysis.

### Data Extraction and Classification

From each included article J.O and M.v.T extracted the following data: authors, journal, study location, publication year, type of intervention, type of exposure (chemical, biological, or both), intervention endpoint, and study population. Thereafter, the articles were classified into four categories depending on the type of intervention: control measures, educational interventions/behavioral changes/training programs (from here behavioral interventions), use of PPE, or policy interventions. Moreover, we classified the articles according to whether the intervention endpoint measured changes in exposure, associated health outcomes, or both. Finally, using the study location data we classified the studies according to the World Bank Atlas method (19): low-income countries (LIC), lower middle-income countries (LMIC), upper middle-income countries (UMIC), and high-income countries (HIC).

### Study Quality Assessment

To assess study quality, we documented for each article the following study design elements which each contribute to enhanced intervention study quality:

1. Planning of intervention, i.e., whether the study data collection and analysis was tailored for the intervention
2. Use of a control group not receiving the intervention
3. Use of randomization of the intervention and control
4. Measurements of pre and post intervention effects.

The overall quality of each study was assessed through calculating a quality score through designating each study one quality point for each fulfilled above listed quality criteria (1 through 4). The quality score thus ranged from a minimum of 0 to a maximum of 4 points.

### Analysis

We analyzed the frequency of articles, study design elements, intervention types, type of exposure, study location (type of country), intervention endpoints, and study quality score of published intervention for the time period 1960–2019, and by 10-year intervals. We additionally analyzed the frequency of publishing journal for the articles. Finally, we assessed the proportion of effective interventions based on whether the author of each included article had concluded the intervention to be successful and/or effective. For this analysis we included both exposure and health outcomes.

## Results

### Article Selection

**Figure 1:**
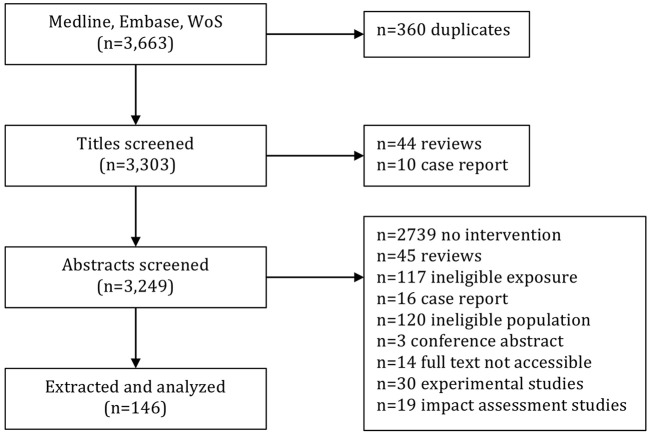
Screening and selection of articles resulting from searches in Medline, Embase, and Web of Science.

Having removed duplicates (*n* = 360) the searches in Medline, Embase, and WoS resulted in a total of 3,303 unique articles subjected to title and abstract screening (Figure 1). During title screening by the first reviewer (J.O), 44 review articles and 10 case reports were excluded. Following, a total of 3,249 abstracts were screened by J.O, which resulted in the exclusion of 2,739 articles which did not describe intervention studies, another 45 review articles, 117 articles analyzing ineligible exposure, 16 case reports, 120 articles with an ineligible study population (e.g., children, residential populations, or animals), 3 conference abstracts, and 14 articles that were not accessible in full text and therefore could not be assessed on their eligibility. The remaining 195 articles were screened by all three reviewers, and resulted in the exclusion of 30 experimental studies and 19 impact assessment studies (Figure 1). This left a total of 146 articles for which data were extracted.

### Characteristics and Examples of Included Articles

The 146 studies published between 1960 and 2019 were performed in a total of 36 countries. The USA served as the most frequent study location for a total of 42 studies, followed by Germany and the Netherlands (11 studies each). The 146 articles were published in 75 different scientific peer-reviewed journals (result not in tables). Based on the conclusions of the authors, 60.2% of included interventions were considered to be effective or successful (results not in tables).

Of the 146 articles on occupational intervention studies, 63 analyzed control measures, 43 behavioral interventions, 28 the use of PPE, and 12 articles evaluated policy interventions (Table 1). For example, regarding control measures Meeker et al. (20) evaluated the effectiveness of local exhaust ventilation (LEV) for a portable abrasive cutter and for tucking point grinders in a bricklayers' training center using a randomized block design. Compared with the control, the LEV unit significantly reduced respirable quartz up to 96% in workers' breathing zone. Moreover, a successful educational intervention aimed to increase the use of PPE to reduce direct pesticide exposure in dairy farmers was evaluated by Perry et al. (21). The three-hour randomly assigned educational sessions (including information on cancer in farmers, and cognitive behavioral strategies to reduce pesticide hazards) showed after six months a significant improvement in the use of PPE and a reduction in the total amount of pesticides used. Moreover, Rajkumar et al. evaluated the effectiveness of a policy intervention to reduce second hand smoke in hospital workers following the introduction of smoking bans in some hospitals in Switzerland in 2010 (22). Using a quasi-experimental design, a reduced cardiovascular risk in terms of heart rate variability and pulse wave velocity was found in the intervention group when compared to the control 3–12 months after the introduction of the ban. Further, Quinlan et al. (23) evaluated the effectiveness of a PPE intervention on exposure to polycyclic aromatic hydrocarbons (PAH) in ten coal liquefaction workers. Using a cross-over design the use of intervention wear (new coverall, shirt, trousers, underwear, socks, and boots) compared to control wear (personal clothing beneath a coverall) resulted after two weeks in significant reductions in workers' absorption and deposition of PAHs.

**Table 1:** Number of articles, intervention types, and intervention endpoints in intervention studies targeting occupational exposure to chemical or biological agents.

	**1960–2019**	**1960–1969**	**1970–1979**	**1980–1989**	**1990–1999**	**2000–2009**	**2010–2019**
Number of articles	146		2	4	19	64	57
**INTERVENTION TYPE**							
Control measure	63		1	3	9	27	23
Behavior/education/training program	43			1	3	19	20
Policy	12				1	7	4
Personal protective equipment	28		1		6	11	10
**INTERVENTION ENDPOINT**							
Exposure	107		1	2	14	44	46
Health outcome	32		1	1	5	15	10

*Articles retrieved in Embase, Medline and Web of Science 1960–2019*.

A majority (73%) of the reviewed articles described intervention studies that measured as endpoint changes in workers' exposures, and about one fifth (22%) measured changes in health outcomes. Few articles (5%) measured both exposures and associated health outcomes in relation to the interventions. In the category of intervention studies measuring exposures, the cluster randomized controlled trial by van Deurssen at al. (24) showed that occupational exposure to quartz was reduced through increased technical control measures in eight companies of the Dutch construction industry. Among concrete drillers, demolishers, and tuck pointers, the reduction of quartz exposure in the intervention group was approximately 30% higher than in the control group. However, the authors concluded that the effect only partially could be attributable to the intervention, as several factors such as changes in work location and abrasiveness of the tasks were not adjusted for. In the category of intervention studies measuring health outcomes, van der Meer et al. (25) showed in a RCT that hand eczema was reported to increase in the intervention group compared to the control group one year after having received an educational intervention. The authors explained the negative result as a possible consequence of an increased awareness of risk factors for hand eczema in the intervention group following the educational sessions, resulting in the intervention group being more likely to report on their hand eczema. Both the impact on exposure and on workers' health resulting from the intervention was investigated by Goodman et al. (26), who examined the impact of smoking bans in workplaces in Ireland in 2004. Using an uncontrolled before-after study design, measurements of the health effects of 81 male bar staff volunteers, and levels of exposure to particulate matter 2.5 μm (PM_2,5_) or smaller, particulate matter 10 μm or smaller (PM_10_), and benzene exposure in 42 pubs were taken. Pre-post comparisons showed a 79% reduction in exhaled breath carbon monoxide, and an 81% reduction in salivary cotinine in the workers', and in the pubs reductions of 83% and 80.2% for PM_2,5_ and benzene, respectively.

### Number of Intervention Studies, Types of Exposure, and Study Location Over Time

When analyzing the articles over time, the number of intervention studies increased with 0 studies published in the time period 1960–1969, 2 studies during 1970–1979, 4 studies during 1980–1989, 19 studies during 1990–1999, and with a noticeable increase in 2000 to 64 studies published during 2000–2009, and 57 studies during 2010–2019 (Table 2). Over the entire time period (1960–2019), 78.1% of the intervention studies targeted chemical exposures, 20.5% biological exposures, and 1.4% targeted both chemical and biological exposures (Table 2). When analyzing types of exposure per 10-year interval no time trend was noticeable (Table 2). Further, the majority (76%) of all interventions studies were carried out in HIC, followed by UMIC (8.2%), and LMIC (6.8%). Few (2.1%) of intervention studies were carried out in LIC, and 6.9% lacked information on study location. When analyzing study location over time no trends were seen (Table 2).

**Table 2:** Relative frequencies of types of exposure, study location, study design elements and study quality of intervention studies targeting occupational exposure to chemical or biological agents.

	**1960–2019**		**1960–1989^a^**		**1990–1999**		**2000–2009**		**2010–2019**	
**Number of articles**	**146**		**6**		**19**		**64**		**57**	
	***n***	**%**	***n***	**%**	***n***	**%**	***n***	**%**	***n***	**%**
**TYPES OF EXPOSURE**										
Chemical	114	78.1	2	33.3	17	89.5	48	75.0	47	82.5
Biological	30	20.5	4	66.7	2	10.5	16	25.0	8	14.0
Both chemical and biological	2	1.4							2	3.5
**STUDY LOCATION**^b^										
LIC	3	2.1							3	5.3
LMIC	10	6.8			2	10.5	2	3.1	6	10.5
UMIC	12	8.2	1	16.7			2	3.1	9	15.8
HIC	111	76.0	5	83.3	14	73.7	55	86.0	37	65.0
Not reported	10	6.9			3	15.8	5	7.8	2	3.5
**STUDY DESIGN ELEMENTS**										
Planned intervention (yes)	126	86.3			16	84.2	55	86.0	51	89.5
Randomization (yes)	23	15.8			1	5.3	14	21.9	8	14.0
Control group (yes)	56	38.4			7	36.8	22	34.4	25	43.9
Pre and post intervention measurements (yes)	125	85.6			16	84.2	52	81.3	52	91.2
**QUALITY SCORE**										
0	3	2.1					3	4.7		
1	26	17.8	2	33.3	5	26.3	12	18.8	7	12.3
2	65	44.5	3	50.0	8	42.1	26	40.6	28	49.1
3	34	23.3	1	16.7	5	26.3	13	20.3	15	26.3
4	18	12.3			1	5.3	10	15.6	7	12.3

Articles retrieved in Embase, Medline and Web of Science 1960–2019. Quality score ranges from 0 to 4.

a *As few (n = 6) articles were published during 1960–1989 the number of articles throughout this entire 30-year interval was used as numerator when calculating percentages of articles for the analysis of time trends during the whole analysis period 1960–2019*.

b *Classification according to the World Bank Atlas Method, 2019 (19). LIC, low-income countries; LMIC, lower middle-income countries; UMIC, upper middle-income countries; HIC, high-income countries*.

### Study Quality

As few (*n* = 6) articles were published during 1960–1989 the number of articles throughout this entire 30-year interval was used as numerator when calculating relative frequencies for the analysis of time trends in article quality during the whole time period 1960–2019 (Table 2). The analysis of study design elements of the 146 included studies showed that 38% applied a control group, 16% randomized (clusters of) workers to an intervention group and a control group, 86% were planned interventions, and 86% evaluated exposure or health outcomes before and after the intervention. Over the whole study period 1960–2019 most studies (45%) had 2 quality points, and only 12% of studies fulfilled all quality criteria, and were thus designated 4 points. Few (2.1%) of the studies were assigned 0 quality points. Over time the quality of studies increased with 0% of studies fulfilling all quality criteria during the 30-year interval 1960–1989, 5% during 1990–1999, 16% during 2000–2009, and 12% during 2010–2019. The percentage of studies with moderate quality (2 points) stayed relatively stable over time (range 40.6–50.0% during 1960–2019), whereas the percentage of those assigned one quality point steadily decreased throughout all analyzed time intervals, with 33.3% during 1960–1989 and 12.3% during 2010–2019 (Table 1).

## Discussion

We reviewed occupational intervention studies specifically focusing on chemical and biological exposures, published in the last six decades 1960–2019. The absolute number and quality of intervention studies was relatively low, whereas we registered a positive trend of increasing number of occupational intervention studies and increasing study quality over time.

This review of occupational intervention studies of chemical and biological exposure is to our knowledge the most comprehensive to date. By searching Medline, Embase, and Web of Science, we searched in the major databases covering life sciences and biomedical literature—a database combination shown to have a high performance (27). Moreover, as indexing of articles is imperfect, we did not solely rely on that relevant articles would be indexed as intervention studies. Instead, we performed our searches using both index terms and search terms qualified by titles/abstracts. Moreover, as some articles analyzing occupational exposures are indexed under the sub-heading “environmental exposure” this search term was also added to the search syntax. The initial combined search retrieval in Embase, Medline, and Web of Science without having introduced the intervention related search terms (search #5 in Supplementary File 1) corresponded to 90,757 articles. The addition of these intervention related search terms reduced the retrieval to 3,303 potentially relevant articles of which almost 85% (*n* = 2,736) did not concern intervention studies, and were retrieved largely as a result of the articles suggesting or mentioning intervention studies in the discussion and conclusion part of the abstract text. The large reduction in article retrieval having introduced the intervention related search terms gives an estimate of the relatively small fraction of articles (146 of 90,757 = 0.2%) of the occupational health literature (concerning our selected exposures) that describe intervention studies. The method of screening the relatively large fraction of non-relevant studies allowed us to capture relevant intervention studies not indexed accordingly.

Despite searching in three large databases this review resulted in a relatively small number of intervention or evaluation studies. Considering that workplace interventions, whether or not specifically designed to reduce exposure, occur very frequently, this result suggest that either very few interventions are properly evaluated on their effectiveness or that such studies are not published in the peer-reviewed literature. Naturally, it was expected that our searches would result in fewer intervention studies than observational studies; generally occupational disease etiology is initially analyzed through several types and iterations of observational studies, and interventional studies are subsequently performed informed by these results, aiming to further prove or break the causal chain between exposure and disease in workers. Still, given the large initial pool of more than 3,000 articles on potentially relevant intervention studies in relation to the actual few (146 of 3303 = 4.4%) eligible number of relevant and eligible intervention studies, there is a potential imbalance between observational studies and subsequent interventions. Despite only covering articles on intervention studies published during 2000 and 2001, a review from 2006 on occupational health interventions also showed few relevant intervention studies (prevalence = 1.7%) in relation to the total number of articles published (28).

Reasons for the relatively low number of occupational intervention studies are manifold, of which some are high costs, and/or logistic hurdles. In situations when such obstacles are present, however, the relatively simple educational intervention of informing workers about their exposure levels and basic measures of protection has been shown to result in significant decreases in exposures, as was demonstrated by Basinas et al. in a farmer population (29). Other explanations for the low number of retrieved studies include the often long-term resource allocation and person-time of exposed workers needed before the effectiveness of an intervention on health outcomes can be properly evaluated (30). The low number is also partly a result of our applied eligibility criteria, excluding experimental studies/laboratory based studies (*n* = 30) (31) and impact assessment studies (*n* = 18) (32). The former were not included as the results of experimental studies have been debated as they may not reflect those obtained under actual working conditions (33). The latter, impact assessment studies, estimate changes in exposure or associated health outcomes under certain hypothesized conditions. As it was the aim of this review to create an overview of occupational intervention studies evaluated under actual working conditions, these studies were excluded.

Over the whole time period 1960–2019 we saw an increase in the number of articles covering occupational interventions on chemical and biological exposures. We particularly noted a stark increase around the year 2000. Although there might be several reason why, it is plausible that the initiative by the NORA program (4) taken in the mid 90ies in the US was a contributing factor to this increase, particularly as the majority of identified studies originated from the US.

Over the time period 1960–2019 we also documented an increase in the quality of included intervention studies. The percentage of studies with the highest quality (4 points) and moderate quality (2 points) both increased over the time period 1960–2019, whereas the percentage of those only assigned 1 point decreased. Despite this increase, overall a majority of studies were assigned only a moderate quality or below (0, 1 or 2 points = 64.4%). Additionally, when examining individual study elements, more than 60 percent of reviewed articles did not incorporated a control group, and close to 85 percent did not randomize workers to an intervention group and a control group. Some other reviews of intervention studies in the OH field also suggest that the quality of evidence resulting from these studies may be deficient. A recent Cochrane review of the effectiveness of behavioral interventions concerning respiratory protective equipment concluded that reviewed studies were of low methodological quality with all included studies judged to have a high risk of bias (34). Further, Robson et al. reviewed articles on the effectiveness of occupational health and safety management system interventions and found that only 1 of 23 articles described an intervention study with a sufficiently high methodological quality (35). Moreover, in the review of OSH interventions by Andersen et al. (6) the authors concluded that the overall quality of evidence was limited and at best moderate, and Roeloef et al. (36) reviewed control strategies for chemical hazards published during 1993–1999 and showed that intervention effectiveness was not consistently evaluated. The general low study quality in our review and in mentioned reviews is possibly related to common difficulties in executing high quality intervention studies in the occupational field. The application of RCTs might be logistically difficult, methodologically challenging, time consuming, expensive, and due to often tight inclusion criteria limited in terms of generalizability (37). If performed however, randomization to intervention and comparison conditions should if plausible be made based on whole worksites (clusters), rather than individual workers, as demonstrated by Lazovich et al. (38). This will prevent contamination due to interaction between workers within the same worksite, which otherwise might bias estimates of the intervention effect. Furthermore, the methodologically fundamental procedure of applying a control group for the evaluation of intervention effectiveness might in some scenarios present problems as it may be considered unethical; to not expose a group of workers with an intervention suggested to have positive health effects might not be accepted by all companies (39). If an intervention study does not apply a control group, conclusions drawn from results might be misleading, as secular trends or sudden changes at the workplace might introduce bias and make it hard to attribute measured effects to the intervention (2). Alternative research designs which partly bypasses mentioned ethical issues associated with applying a control group comprise for example the stepped-wedge randomized trial or the multiple baseline approach, in which the intervention is implemented in stages across the whole workforce (39, 40). Also, studies of “natural” experiments, i.e. studies where the researcher has no influence over the implementation of the intervention, can provide very useful data on the effectiveness of the intervention. However, lack of a baseline and/or control group will limit the value of the results from this type of study. To partly address mentioned ethical considerations, also the control group might receive some degree of intervention. This might however limit the generalizability of the study results. In The Minnesota Wood Dust Study the PRECEDE-PROCEDE model was used to develop an intervention for reducing dust exposure that was acceptable to the woodworking industry (41, 42). Businesses in the intervention group and the control group both received written recommendations to improve the control of dust. The intervention group additionally received technical assistance and worker training. The authors partly attributed the lack of any statistically significant reduction in dust concentrations between the intervention and control to the degree of intervention that also had been performed in the control group. Thus, despite the application of a control group investigated intervention effects might be diluted, which weakens external study validity.

This review has its limitations. Due to time and budget constraints it was not possible for the 2^nd^ and 3^rd^ reviewer to independently assess the eligibility of all initial 3,303 articles. Nevertheless, after the 1^st^ reviewer had excluded clearly ineligible articles (most notably articles describing studies that solely mentioned the word “intervention” in the abstract), potentially eligible articles were assessed by all three reviewers. Moreover, our search syntax was based on terms reflecting some common occupational chemical and biological exposure. However, our search syntax did not include specific occupational diseases associated with eligible exposures. Thus, some articles describing intervention studies focusing on the actual occupational disease without describing our selected exposures might not be part of this review.

Despite an upward trend over time in the number and quality of occupational interventions studies, more work is needed to generate stronger evidence of the effectiveness of different types of occupational interventions to further outline what works and what does not work, preventing that resources are wasted on implementing ineffective or unnecessary interventions. This is further underlined by the fact that only 60% of the reviewed interventions were considered effective or successful.

Occupational exposure to chemical and biological agents is still causing excessive disease in workforce worldwide, including Europe and North America. It is perhaps surprising that despite that, as occupational health professionals, we constantly emphasize the need for preventative action in the workplace to reduce occupational ill health, we develop very little sound evidence on the effectiveness of interventions in the workplace. Although sensible best practice advice such as provided in COSHH essentials may be used to address exposures that are excessively high, reducing exposures further will require more sophisticated evidence-based interventions to ensure that trends in declining exposures and negative health impact continue and occur globally. It is therefore important to apply properly designed occupational intervention studies enabling the evaluation of intervention effectiveness to further expand the evidence on (cost-)effective interventions. As argued by Cherrie et al. (43), the occupational field ought to strive toward acceptable, and not just tolerable, occupational exposure. This is to a large extent achieved through the continuous improvement of the methodology and effectiveness of occupational intervention studies, ensuring the reduction of harmful exposure and related health risks globally in occupational populations.

## Conclusion

Despite an increase in the frequency and quality of intervention studies targeting exposure to chemicals and biological agents during the last six decades, the absolute number and quality of studies is still low. Occupational exposure to chemical and biological agents is still causing excessive disease in workforce worldwide. A persistent reduction of exposures will require more sophisticated and effective evidence-based interventions to ensure that trends of reducing exposures and negative health impact continue and occur globally. Consequently, we must increase the number of high quality studies implementing and evaluating several types of interventions and settings to improve our understanding of their (cost-)effectiveness and transferability.

## Author Contributions

JO performed the majority of the article screening, the article analysis, the manuscript compilation, and extracted the data together with MT. HK and MT performed article screening and selection, and gave feedback on the manuscript.

### Conflict of Interest

MT was invited to present a keynote lecture at the EPICOH 2019 conference in Wellington, New Zealand. Travel and accommodation was supported by the conference organizers. The remaining authors declare that the research was conducted in the absence of any commercial or financial relationships that could be construed as a potential conflict of interest.
