# Supplementary material for: Interventions to Reduce Exposures in the Workplace: A Systematic Review of Intervention Studies Over Six Decades, 1960–2019
Source: Front Public Health. 2020 Mar 9;8:67. doi: 10.3389/fpubh.2020.00067 (PMC7075246; doi:10.3389/fpubh.2020.00067)
Supplement: Supplementary file 1 [file Data_Sheet_1.pdf]

frontiers

Supplementary file 1. Search terms.

**Supplementary file 1.** Search terms used in Embase to target occupational intervention studies on exposure to chemical and biological agents published 1960-2019. For searches in Medline and Web of Science the same search terms were applied using database-specific syntax.	
	**Exposures**
#1	((‘chemic*’:ti,ab OR 'organic dust':ti,ab OR 'dust':ti,ab OR ‘biologic* dust’:ti,ab OR 'fume*':ti,ab OR 'vapor*':ti,ab OR 'fiber*':ti,ab OR 'microorganism*':ti,ab not ‘radiation’:ti,ab) AND ‘exposure’:ti,ab) AND (occupation*:ti,ab OR work*:ti,ab OR job*:ti,ab OR employ*:ti,ab OR factory:ti,ab OR industr*:ti,ab OR ‘person* protect* equip*’:ti,ab)
#2	'occupational safety' OR 'chemical exposure'/exp OR 'occupational exposure'/exp OR 'organic dust'/exp OR 'dust'/exp OR ‘biological dust’/exp OR 'fume'/exp OR 'vapor'/exp OR 'fiber'/exp OR 'microorganism'/exp) AND (occupation*:ti,ab OR work*:ti,ab OR job*:ti,ab OR employ*:ti,ab OR factory:ti,ab OR industr*:ti,ab OR ‘person* protect* equip*’:ti,ab)
#3	((‘chemic*’:ti,ab OR 'organic dust':ti,ab OR 'dust':ti,ab OR ‘biologic* dust’ OR 'fume*':ti,ab OR 'vapor*':ti,ab OR 'fiber*':ti,ab OR 'microorganism*':ti,ab not ‘radiation’:ti,ab) AND ‘exposure’:ti,ab) AND ('environmental exposure'/exp) AND (occupation*:ti,ab OR work*:ti,ab OR job*:ti,ab OR employ*:ti,ab OR factory:ti,ab OR industr*:ti,ab OR ‘person* protect* equip*’:ti,ab)
#4	#1 OR #2 OR #3
	**Intervention types**
#5	(('intervention'/exp OR 'intervention study'/exp OR 'control measure*':ti,ab OR intervention*:ti,ab OR ‘person* protect* equip*’:ti,ab) OR (('random*' NEAR/5 'control*'):ti,ab) OR (('control*' NEAR/5 'trial*'):ti,ab)
#6	#4 AND #5
	**Excluded outcomes**
#7	#6 NOT ('musculoskeletal system'/exp OR 'mental disease'/exp OR 'stress'/exp OR 'ergonomics'/exp)

#8	#7 AND ([danish]/lim OR [dutch]/lim OR [english]/lim OR [french]/lim OR [german]/lim OR [norwegian]/lim OR [spanish]/lim OR [swedish]/lim) AND [1-1-1960]/sd NOT [1-1-2020]/sd
